# An open source multistep model to predict mutagenicity from statistical analysis and relevant structural alerts

**DOI:** 10.1186/1752-153X-4-S1-S2

**Published:** 2010-07-29

**Authors:** Thomas Ferrari, Giuseppina Gini

**Affiliations:** 1 Department of Electronics and Information (DEI), Politecnico di Milano via Ponzio, 34/5 - 20133 Milano, Italy

## Abstract

**Background:**

Mutagenicity is the capability of a substance to cause genetic mutations. This property is of high public concern because it has a close relationship with carcinogenicity and potentially with reproductive toxicity. Experimentally, mutagenicity can be assessed by the *Ames test *on Salmonella with an estimated experimental reproducibility of 85%; this intrinsic limitation of the *in vitro *test, along with the need for faster and cheaper alternatives, opens the road to other types of assessment methods, such as *in silico *structure-activity prediction models.

A widely used method checks for the presence of known *structural alerts *for mutagenicity. However the presence of such alerts alone is not a definitive method to prove the mutagenicity of a compound towards Salmonella, since other parts of the molecule can influence and potentially change the classification. Hence statistically based methods will be proposed, with the final objective to obtain a cascade of modeling steps with custom-made properties, such as the reduction of *false negatives*.

**Results:**

A cascade model has been developed and validated on a large public set of molecular structures and their associated Salmonella mutagenicity outcome. The first step consists in the derivation of a statistical model and mutagenicity prediction, followed by further checks for specific structural alerts in the "safe" subset of the prediction outcome space. In terms of accuracy (i.e., overall correct predictions of both negative and positives), the obtained model approached the 85% reproducibility of the experimental mutagenicity *Ames test*.

**Conclusions:**

The model and the documentation for regulatory purposes are freely available on the CAESAR website. The input is simply a file of molecular structures and the output is the classification result.

## Background

In our everyday life we have to deal with an ever increasing number of new and different chemical compounds, such as food colourings and preservatives, drugs, dyes for clothes and ordinary objects, pesticides and many others: at present the number of registered chemicals is estimated at 28 million. It is well recognised that uncontrolled proliferation of new chemicals may pose risks to the environment and people; hence, their potential toxicity has to be considered. Biologically active chemicals interact with biomolecules, triggering specific mechanisms, such as the activation of an enzyme cascade or the opening of an ion channel, which lead to a biological response. These mechanisms, determined by the chemical properties, are unfortunately largely unknown; thus, toxicity tests are needed.

Mutagenic toxicity, also called mutagenicity, can be assessed by various test systems. It is a property of high public concern because it has a close relationship with carcinogenicity and, in the case of germ cell mutations, with reproductive toxicity [1]. For assessing the potential of a chemical to be toxic, a significant breakthrough was the creation of cheap and short-term alternatives to the rodent bioassay, the main tool of the research on chemical carcinogens. With this intent, Bruce Ames created a series of genetically engineered Salmonella Typhimurium bacterial strains, each strain being sensitive to a specific class of chemical carcinogens [2]. As discussed in other papers [3], the estimated inter-laboratory reproducibility of this *in vitro *test is about 85%. This observation will be taken into account in the conclusive discussion. Alongside classical experiments for assessing toxicity, the use of computational tools is gaining more and more interest in the scientific community and in the industrial world as accompaniment to or replacement of existing techniques. Whereas animal tests are very expensive and time consuming, high throughput computational approaches, otherwise known as *in silico *models, are broadening the horizons of experimental sciences: with increasing sophistication of such models, we are increasingly moving from experiments to simulations [4].

In the *in silico *branch of the hazard estimation field, a common technique consists of putting into practice the Structure-Activity Relationship principle in either a qualitative or a quantitative way. The qualitative approach (SAR) may simply consist of the automated detection, in a structural representation of the compound, of particular fragments known to be a main determinant of the toxic property under investigation. In the mutagenicity/carcinogenicity domain, the key contribution in the definition of such toxicophores comes from Ashby's studies in the 80s [5]. Grounding his work on the electrophilicity theory of chemical carcinogenesis developed by Miller and Miller [6,7], which correlates the electrophiles presence (like halogenated aliphatic or aromatic nitro substructures) to genotoxic carcinogenicity, Ashby compiled a list of 19 *structural alerts *for DNA reactivity. Subsequent efforts have built on the knowledge collected by Ashby to derive more specific rules, such as reported in the more recent work of Kazius and coworkers [8] whereby the cognition of the mechanism of action is joined to statistical criteria.

In other cases, when the physicochemical properties or structural information of chemicals and their potency are numerically quantified, it is possible to search for a mathematical correlation between the chemical's properties and its biological activity, i.e., the quantitative (QSAR) approach. These computed properties are referred to as molecular descriptors [9] and their computation can be carried out by many software packages (mainly commercial) starting from the structural representation, even for those chemicals not yet synthesised. Therefore, with a machine learning algorithm, the study of interactions between molecules and living organisms can be approached like a data mining problem.

How can these techniques be made more suitable for regulatory purposes? An answer is to address the reduction of *false negatives *with special care, i.e., those hazardous compounds predicted incorrectly as safe. There are various tricks to implement such enhancement simply by skewing the model and making it more sensitive to toxicity, but all attempts in this direction will unavoidably cost a marked increase in *false positive *rate as the *false negative *rate slightly decrease. In this context we propose the idea of a trained QSAR classifier supervised by a SAR layer that incorporates coded human knowledge. The aim is to refine the good separation between classes supplied by the statistical model, not by introducing a perturbation in its optimality, but by equipping it with a knowledge-based facility to minutely identify misclassified toxic substances.

In the following sections this paradigm is implemented for modeling the well-studied endpoint of mutagenicity. Initially, a classifier is trained on more than four thousand molecules by data mining a selection of calculated descriptors; in a second step, the relative knowledge to complement its practice is extracted from a collection of well-known *structural alerts*. The resulting model is validated and implemented to be freely available through the portal of the CAESAR project http://www.caesar-project.eu[10].

## Results and discussion

**Figure 1:**
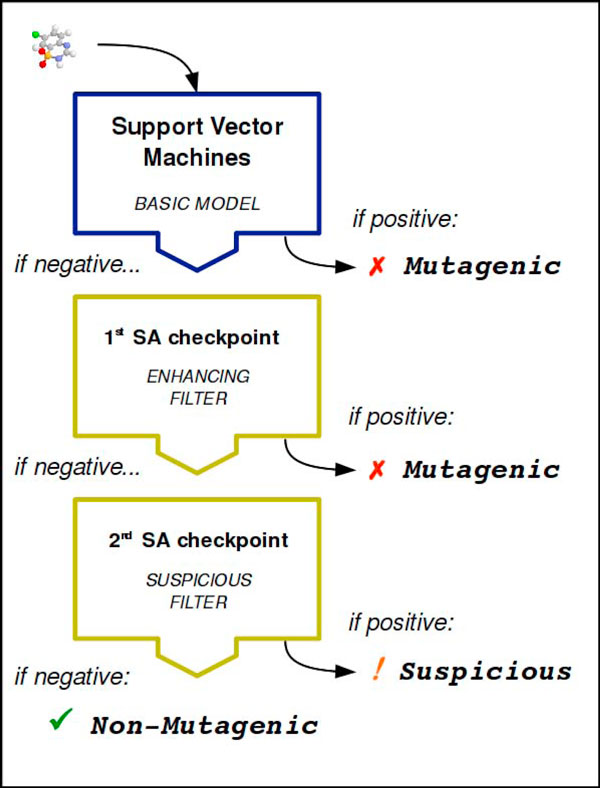
**The architecture of the integrated mutagenicity model: cascading filters**.

To achieve a tool more suitable for regulatory purposes, a mutagenicity classifier has been arranged integrating two different techniques: a machine learning algorithm from the Support Vector Machines (SVM) collection, to build an early model with the best statistical accuracy, then an ad hoc expert system based on known *structural alerts *(SAs), tailored to refine its predictions. The purpose is to prevent hazardous molecules misclassified in first instance (*false negatives*) from being labelled as safe. The resultant classifier can be presented as a cascading filters system (see Figure 1): compounds evaluated as positive by SVM are immediately labelled *mutagenic*, whereas the presumed negatives are further sifted through two consecutive checkpoints for SAs with rising sensitivity. The first checkpoint (12 SAs) has the chance to enhance the prediction accuracy by attempting a precise isolation of potential *false negatives *(FNs); the second checkpoint (4 SAs) proceeds with a more drastic (but more prudent) FNs removal, as much as this doesn't noticeably downgrade the original accuracy by generating too many *false positives *(FPs) as well. To reinforce this distinction, compounds filtered out by the first checkpoint are labelled *mutagenic *while those filtered out by the second checkpoint are labelled *suspicious: *this label is a warning that denotes a candidate mutagen, since it has fired a SA with low specificity. Unaffected compounds that pass through both checkpoints are finally labelled *non-mutagenic*.

Table 1 and Table 2 present the confusion matrices on test and training sets with the three distinct outputs of the model. The choice for the final binary classification of suspicious compounds is left to the end-user, depending on his/her priority to either obtain accurate results, by considering them as non-mutagens, or to obtain a more prudent classification minimising FNs, by considering them as mutagens. This leads to two different statistical results, according to the actual choice either to maximise accuracy or to minimise FNs.

**Table 1:** Confusion matrix of the mutagenicity integrated model on the test set (837 chemical compounds).

Test set (837 chemicals)	Mutagenic predictions	Non-mutagenic predictions	Suspicious predictions
Mutagens	403	48	14
Non-mutagens	88	268	16

The "suspicious" label marks potential mutagens picked out from the "non-mutagenic" predictions.

**Table 2:** Confusion matrix of mutagenicity integrated model on the training set (3367 chemical compounds).

Training set (3367 chemicals)	Mutagenic predictions	Non-mutagenic predictions	Suspicious predictions	Unpredicted compounds
Mutagens	1798	69	15	1
Non-mutagens	169	1239	76	0

The low number of true positives in the suspicious set, if compared with the test set confusion matrix (cf. Table 1), is due to the very small number of real mutagens in the "non-mutagenic" predictions on the training set. The unpredicted structure was not processed by the CDK library.

**Figure 2:**
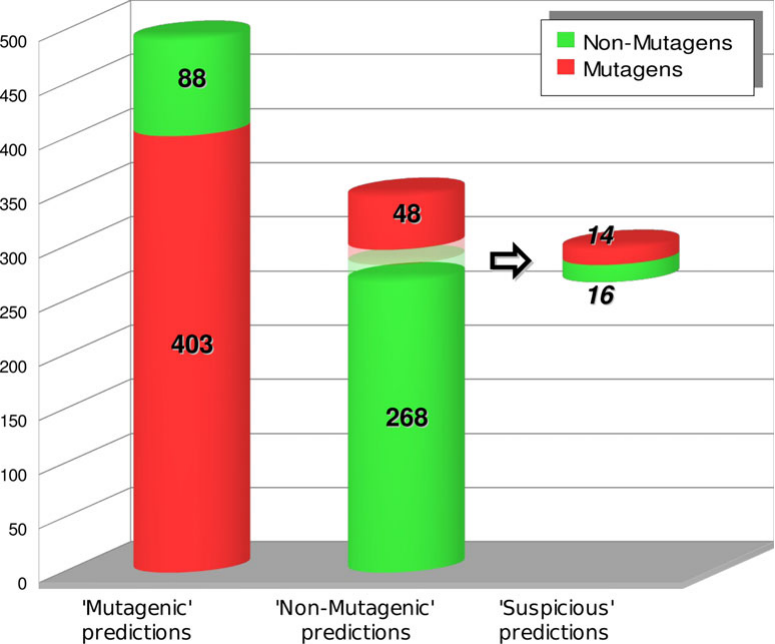
**Graph view of the final model prediction on the test set (837 chemicals)**. This representation highlights how the suspicious rules set can extract the most suspect compounds from safe predictions with a good accuracy, if related to the very low number of real mutagens still present.

In Table 3 the statistics of the integrated model are compared on the same test set to those of its two single components: the SVM classifier and the Benigni/Bossa rulebase [11], i.e., the set of 30 rules for mutagenicity from which the SAs used in the final model have been derived. As can be seen, the final model globally outperforms its antecedents in both its possible implementations. With regard to the "max accuracy" implementation, both sensitivity (87%) and accuracy (82%) are improved (respectively: +3% and +1%), with respect to the original SVM classifier, by the removal of 16% of FNs. Conversely, with the "min FNs" policy an impressive 35% reduction in FNs number has boosted sensitivity to 90%, keeping accuracy almost unaltered at the cost of only a slight decrement in specificity (72%). A visual representation of prediction ability of the combined model on the test set is illustrated in Figure 2.

**Table 3:** Compared statistics, on the test set, between the integrated model and its single components: the SVM statistical model and the Benigni/Bossa structural alerts set for mutagenicity.

Test set (837 chemicals)	Benigni/Bossa rulebase	SVM classifier	Integrated model (max accuracy)	Integrated model (min FNs)
accuracy:	78.3%	81.2%	82.1%	81.8%
sensitivity:	86%	84.1%	86.7%	89.7%
specificity:	69.6%	77.7%	76.3%	72%
*SAs:*	*30*		*12*	*16*
*descriptors:*		*25*	*25*	*25*

To evaluate the structural alerts, the "official" (commissioned by JRC) Toxtree v. 1.60 implementation of the Benigni/Bossa rulebase has been used.

## Experimental

### The dataset

For the development and the validation of the model, the *Bursi Mutagenicity Dataset *has been used. It is a large data set, containing *4337 *molecular structures with the relative Ames test results, described in the previously mentioned paper by Kazius et al. [8].

Such data set has been further verified within the CAESAR project to improve its accuracy and the robustness of the consequent model; in particular, each chemical structure was checked and this data quality control procedure introduced an overall reduction of the number of molecular structures in the set: the resulting data set consists now of *4204 *compounds, *2348 *classified as mutagenic and *1856 *classified as non-mutagenic by Ames test. To provide concrete basis for validation, the data set was split into a training set and a test set following a stratification criterion in order to make sure that each subset would approximately cover all major functional groups as well as all major features of the chemical domain of the total compound set. The training set used for the development of the model consists of 80% of the entire data set (*3367 *compounds), while the other 20% (*837 *compounds) has been left for testing. See additional file 1: *MutagenicityDataset_4204.csv*.

For every chemical in the data set, a great number of molecular descriptors was initially calculated by MDL QSAR commercial software [12]. Then, a subset of 27 descriptors was selected by using the tools provided by the Weka 3.5.8 environment for data mining [13]. The BestFirst algorithm was mainly used as bidirectional search method in the descriptors subsets, using as subset evaluator the 5-folds cross-validation score on the training set (in short: BestFirst algorithm searches the space of attribute subsets by greedy hill climbing, considering all possible single attribute additions or/and deletions at a given point, with a backtracking facility to explore also non-improving nodes).

Finally, to allow a free utilisation of the model, the needed descriptors were re-implemented from scratch with the CDK 1.2.3 open source Java library. This passage caused slight alterations in the data and two descriptors have been removed, as one turned out to be a duplicate and the other one was too ambiguous for implementation. Moreover, a MDL descriptor (*LogP*) has been replaced by its Dragon [14] equivalent (*ALOGP*) for which a special license has been acquired. This perturbation did not significantly affect the overall behaviour, but it made possible to compute on line the descriptors by the CAESAR web application, starting from the structural representation of compounds (i.e., SDF file or SMILES [15] ASCII string).

Of *25 *total descriptors used in the final implementation, *4 *are global descriptors: *Gmin*, the minimum E-state value for all the atoms in the molecule [16]; *idwbar*, the Bonchev-Trinajstic mean information content based on the distribution of distances in the graph; *ALOGP*, the Ghose-Crippen octanol water coefficient [9,17] and *nrings*, the number of rings in the molecular graph (the cyclomatic number: the smallest number of bonds which must be removed such that no ring remains). All the others are simple atom type counts, namely the count of some type of e-state fragment. In other words, they are the counts of small 2D fragments composed of an element and its bonding environment (for the propane example is the count of all -CH3 groups in a molecule).

In order to allow it to be used by the machine learning algorithm, the descriptors matrix in the training set was normalised by simply dividing each descriptor column by its maximum absolute value. See additional file 2 (*25descriptors_Legend.xls*) for the selected descriptors list and description and additional file 3 (*25descriptors_Dataset.csv*) for the complete calculated descriptor dataset.

### The C-SVC classification algorithm

The machine learning algorithm chosen comes from the SVM family, a collection of supervised learning methods for classification and regression with well-founded basis in statistical learning theory. These learning methods are already successfully used in many application domains such as pattern recognition [18], drug design [19] and QSAR [20]. In particular, the *C*-Support Vector Classification algorithm used to build the model is a method originally proposed by Vapnik [21] and later extended for nonlinear classification at AT&T Bell Labs [22]. In a few words, the optimisation problem solved by the algorithm consists of finding the *maximum margin separator hyperplane *in the input space. This is the hyperplane that separates the two classes in the space of descriptors, minimising the classification error and, at the same time, maximising the *margin *(i.e. the distances from the hyperplane to the closest samples of both classes, called *support vectors*): the idea is to find out the best trade off between accuracy and generalisation. The result is a linear classifier, but SVM can still use linear models to implement nonlinear class boundaries thanks to a nonlinear mapping easily implemented with the "kernel trick" [23]. In other words, the input space is mapped into a higher dimensional space by a nonlinear function, and a linear model constructed in the new space can represent a nonlinear decision boundary in the original space. The choice of the kernel function of the algorithm fell on Radial Basis Function (RBF), as shown in previous experiment on the same chemical set [20].

A complete but smart environment to develop SVM models is provided by the open source LibSVM library [24], containing C++ and Java implementation of SVM algorithms with high-level interfaces (Python, Weka and more) and equipped with some useful tools. Within this environment, building a good model for the mutagenicity classification issue is a straightforward task [25].

### The multi-step model

The search for the optimal parameterisation of the SVM classifier concerning this specific task was fully automated by one of the scripts (*grid.py*) included in the LibSVM 2.89 library. With this tool it is possible to perform an almost exhaustive grid-search in the 2-dimensional parameters space of the classification algorithm, using as evaluation criterion the cross-validation score on a given data set. The best assignment found by such calibration procedure by 10-folds-cross-validating the training set was (C, g) = (8,16). With these parameters a classifier was trained and its prediction ability evaluated on the untouched test set, normalised with the same scale factors used for the training set. This provided a basic model with very good performance.

In order to enhance the classification ability it would be a sound idea to perform a further scan for known *structural alerts *(SAs) for mutagenicity on those compounds predicted non-mutagenic by the SVM model. But in practice, since the subset of compounds under evaluation has already been cleaned from the majority of mutagens by another classifier, the indiscriminate search for SAs can introduce more inaccuracies than benefits. In fact, while the potential intrinsic error rate of every rule based on SAs remains unaltered, since all the non-mutagens should be still present (that means new FPs generated), the number of possible hits (caught FNs) drastically decreases because just a few mutagens are left. Hence, a selection is needed to extract just the necessary knowledge to complement the training of the machine learning algorithm. This can be achieved by a subset of SAs skilled in filtering right the mutagens that are potentially subject to misclassification by the SVM model.

Thanks to such a large data set, the selection of such relevant SAs can be carried out looking at the predictions obtained by cross-validating the SVM classifier on the training set; they are representative of its general prediction ability, so a filter fixing inaccuracies of such predictions will probably provide even for defects of the real model.

With this objective in mind, we considered the collection of 30 SAs for mutagenicity derived by Benigni and Bossa from several literature sources [5,8,26,27] and arranged them in a rule base as exhaustive and non-redundant as possible [11]. Once evaluated on the cross-validated predictions, the analysis of the behaviour of these rules (again, implemented with the CDK library) determined two sets of SAs helpful in different ways. The first set of "enhancing" rules (12 SAs), showed a balance of more FNs caught than FPs generated; the second set is characterized by "suspicious" rules (4 SAs), still showing a remarkable FNs removal power but also a higher misclassification rate. See additional file 4*SA.pdf *to view the selected SAs. During this selection procedure, the performance on the data set of rules with a very low number of compounds involved was considered not reliable and its behaviour in the original paper was verified; in this case we considered safe to be used only those rules with a nominal FP rate of 0%, in order to prevent unexpected FPs proliferation. Their supposed capacity to refine the SVM classifier prediction ability was confirmed by the proof on the test set. The output predictions are summarised in additional file 5: CAESAR_Predictions.csv.

## Conclusions

In terms of accuracy, the proposed model can get very close to the 85% of reliability of the experimental mutagenicity *Ames test*, as mentioned in the introduction of this paper. Since what the model is predicting is the outcome of the experimental test itself, and not the real mutagenic property, searching for an higher precision can be misleading. A 100% of accuracy rate would involve a real correctness equal to the reliability of such test: the model would be learning the experimental error as well.

Besides this, the model has the more important feature of being tailored for regulatory purposes with a *false negative *removal tool: on the test set, a satisfactory accuracy (82%) is preserved while the false negative rate is reduced from 16% to 10%.

The excellent results achieved by this integrated model on the mutagenicity prediction issue open the way to a new era of hybrid models, customisable to meet different requirements. Currently, no attempts have been made to apply this method to other endpoints.

## List of abbreviations used

SAR: Structure-Activity Relationship; QSAR: Quantitative Structure-Activity Relationship; SVM: Support Vector Machines; SA: Structural Alerts; FN: False Negative; FP: False Positive

## Competing interests

The authors declare that they have no competing interests.

## Supplementary Material

Additional file 1 **The pruned Bursi Mutagenicity Dataset**. A collection of 4204 chemical structures with the relative Ames test result. Click here for file

Additional file 2 **Selected descriptors**. The list and the definitions of the 25 selected molecular descriptors. Use Excel to properly view the atom-type bond representations. Click here for file

Additional file 3 **Descriptors dataset**. The calculated value (by the CAESAR web application) of the used descriptors for all the chemical structures in the data set. Structure with "Mol ID" #311 was not processed by the CDK library. Click here for file

Additional file 4 **Selected *structural alerts***. The two subsets of *structural alerts *selected from the Benigni/Bossa rulebase (3 pages). Click here for file

Additional file 5 **Model outcome**. Data for each compound: molecule ID, CAS number, experimental Ames test, predicted class, author of prediction (i.e., SVM classifier, 1st SAs check, 2nd SAs chek), set to which the compound belong (i.e., training set, test set). Molecule #311 was not processed by the CDK library. Click here for file
