# Supplementary material for: An open source multistep model to predict mutagenicity from statistical analysis and relevant structural alerts
Source: Chem Cent J. 2010 Jul 29;4(Suppl 1):S2. doi: 10.1186/1752-153X-4-S1-S2 (PMC2913329; doi:10.1186/1752-153X-4-S1-S2)
Supplement: Additional file 4 — Selected structural alerts. The two subsets of structural alerts selected from the Benigni/Bossa rulebase (3 pages). [file 1752-153X-4-S1-S2-S4.pdf]

1st SAs RULESET

Structural Alert	SMARTS or details
**SA_1: Acyl halides**<br>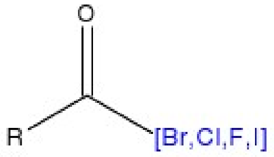<br>R = any atom/group, except OH, SH	`[!$([OH1,SH1])]C(=O)[Br,Cl,F,I]`
**SA_6: Propiolactones or propiosultones**<br>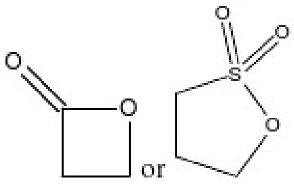	`[O,S]=C1[O,S]CC1`<br>OR<br>`O=S1(=O)(CCCO1)`
**SA_12: Quinones**<br>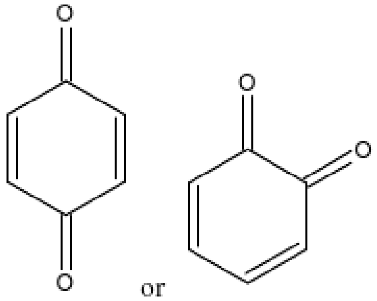<br>R = any atom/group	`O=[#6]1[#6]=,:[#6][#6](=O)[#6]=,:[#6]1`<br>OR<br>`O=[#6]1[#6]=,:[#6][#6]=,:[#6][#6]1(=O)`
**SA_13: Hydrazine**<br>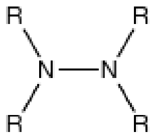	`[N+0]!@;-[N+0](=[!O;!N])`<br>OR<br>`[N+0]([#1,*])!@;-[N+0]([#1,*])`

1st SAs RULESET	
**Structural Alert**	**SMARTS or details**
**SA_14: Aliphatic azo and azoxy**<br>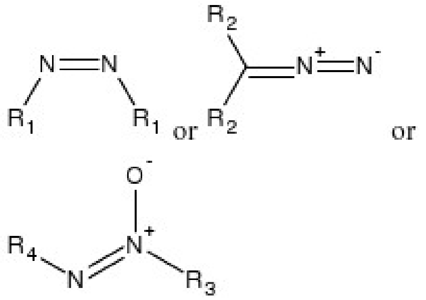<br>R1= Aliphatic carbon or hydrogen<br>R2, R3 = Any atom/group<br>R4 = Aliphatic carbon	`[C,#1]N=[NX2][C,#1]`<br>OR<br>`[$(C=[N+]=[N-]);!$(C=[N+]=[N-]=N);!$(C=[N+]=[N-]N)]`<br>OR<br>`C=[$(N=N);!$(N=N=N);!$(N=NN)]`<br>OR<br>`CN=NO`
**SA_16: alkyl carbamate and thiocarbamate**<br>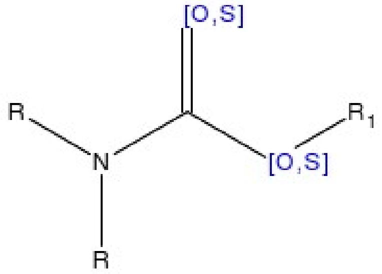<br>R = Aliphatic carbon or hydrogen<br>R1 = Aliphatic carbon	`[NX3]([CX4,#1])([CX4,#1])C(=[O,S])[O,S][CX4]`
**SA_18: Polycyclic Aromatic Hydrocarbons**	Three or more fused rings, not heteroaromatic

1st SAs RULESET	
**Structural Alert**	**SMARTS or details**
**SA_21: alkyl and aryl N-nitroso groups**<br>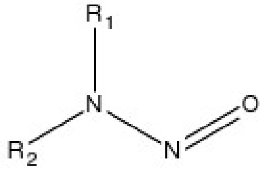<br>R1= Aliphatic or aromatic carbon,<br>R2= Any atom/group	`[C,c]N[NX2;v3]=O`
**SA_22: azide and triazene groups**<br>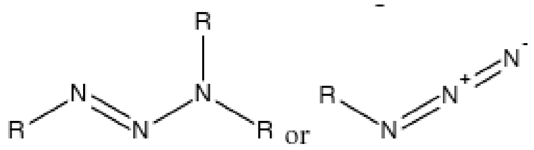<br>R= Any atom/group	`[N]=[N]-[N]`<br>OR<br>`[N]=[N]=[N]`
**SA_25: aromatic nitroso group**<br>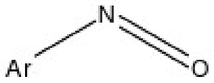<br>Ar = Any aromatic/heteroaromatic ring	
**SA_28bis: Aromatic mono- and dialkylamine**<br>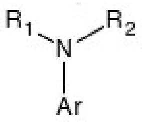<br>Ar = Any aromatic/heteroaromatic ring<br>R1 = Hydrogen, methyl, ethyl<br>R2 = Methyl, ethyl	• Chemicals with ortho-disubstitution, or with an ortho carboxylic acid substituent are excluded.<br>• Chemicals with a sulfonic acid group (- SO3H) on the same ring of the amino group are excluded .
**SA_29: Aromatic diazo**<br>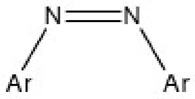<br>Ar = Any aromatic/heteroaromatic ring	• Chemicals with a sulfonic acid group (-SO3H) on both rings linked to the diazo group are excluded.

<table>
<tr><th colspan="2">2nd SAs RULESET</th></tr>
<tr><th>Structural Alert</th><th>SMARTS or details</th></tr>
<tr><td>SA_7:Epoxides and aziridines
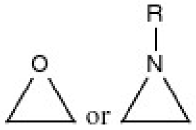

R = any atom/group</td><td><code>C1[O,N]C1</code></td></tr>
<tr><td>SA_8: Aliphatic halogens
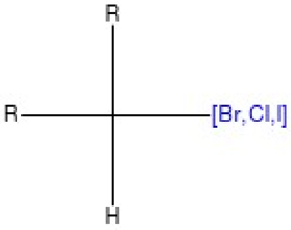

R = any atom/group</td><td></td></tr>
<tr><td>SA_19: Heterocyclic Polycyclic Aromatic
Hydrocarbons</td><td>Three or more fused rings, heteroaromatic</td></tr>
<tr><td>SA_27: Nitro-aromatic
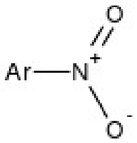

Ar = Any aromatic/heteroaromatic ring</td><td>Chemicals with ortho-disubstitution, or with an ortho carboxylic acid substituent are excluded.
Chemicals with a sulfonic acid group (-SO3H) on the same ring of the nitro group are excluded .</td></tr>
</table>
